# Innate Immunity and Synovitis: Key Players in Osteoarthritis Progression

**DOI:** 10.3390/ijms252212082

**Published:** 2024-11-11

**Authors:** Veronica Panichi, Silvia Costantini, Merimma Grasso, Carla Renata Arciola, Paolo Dolzani

**Affiliations:** 1 Laboratory of Immunorheumatology and Tissue Regeneration, IRCCS Istituto Ortopedico Rizzoli, 40136 Bologna, Italy; paolo.dolzani@ior.it; 2 Department of Medical and Surgical Sciences (DIMEC), Alma Mater Studiorum University of Bologna, 40136 Bologna, Italy; silvia.costantini6@studio.unibo.it (S.C.); merimma.grasso@studio.unibo.it (M.G.); 3 Laboratory of Immunorheumatology and Tissue Regeneration, Laboratory of Pathology of Implant Infections, IRCCS Istituto Ortopedico Rizzoli, 40136 Bologna, Italy

**Keywords:** osteoarthritis, osteoarthritis pathogenesis, innate immunity, innate immunity cells, synovium, synovitis, inflammation, low-grade inflammation, inflammaging, loss of chondrocyte homeostasis

## Abstract

Osteoarthritis (OA) is a chronic progressive disease of the joint. Although representing the most frequent cause of disability in the elderly, OA remains partly obscure in its pathogenic mechanisms and is still the orphan of resolutive therapies. The concept of what was once considered a “wear and tear” of articular cartilage is now that of an inflammation-related disease that affects over time the whole joint. The attention is increasingly focused on the synovium. Even from the earliest clinical stages, synovial inflammation (or synovitis) is a crucial factor involved in OA progression and a major player in pain onset. The release of inflammatory molecules in the synovium mediates disease progression and worsening of clinical features. The activation of synovial tissue-resident cells recalls innate immunity cells from the bloodstream, creating a proinflammatory milieu that fuels and maintains a damaging condition of low-grade inflammation in the joint. In such a context, cellular and molecular inflammatory behaviors in the synovium could be the *primum movens* of the structural and functional alterations of the whole joint. This paper focuses on and discusses the involvement of innate immunity cells in synovitis and their role in the progression of OA.

## 1. Background

Osteoarthritis (OA) is the most common joint disease in older adults. It affects more than five hundred million people worldwide, 60% of whom are over the age of fifty-five. Due to population changes (aging and obesity), the prevalence of OA is expected to double by 2050 [1,2]. Although osteoarthritis can affect any joint or even multiple joints [3], knee OA (KOA) is the most prevalent and debilitating form, accounting for up to 65% of total OA cases.

Several risk factors are associated with OA, in particular: age, which is the main one, female sex, genetic predisposition, obesity, diet, but also injuries and abnormal loading of the joints due to malalignment [4]. Depending on the associated risk factor and phenotype, several subgroups of OA have been. The first subgroup defined by mechanistic phenotypes, characterized by the involvement of molecular mechanisms that lead to OA development (such as genetic, age-related, and metabolic OA,), is the most referred to for OA subtype definition. Two more subgroups, prognostic phenotypes and response to therapy phenotypes, use different approaches for categorization based on possible clinical approaches or outcome predictors to specific therapeutic approaches [5,6]. Recently, obesity is gaining a prominent role in OA, especially in relation to metabolic syndrome, due to the increase of these comorbidities in the population in developed countries. The merging of OA and metabolic syndrome has sparked the definition of a new disease subtype known as metabolic OA. The growing prevalence of this subtype sparks concerns due to the possible related early-onset of OA in the affected population [7,8].

Common features of OA include stiffness, swelling, pain, and, ultimately, impairment of mobility in the affected joint [9,10]. Indeed, OA is the leading cause of motor disability worldwide [10]. Clinical guidelines for symptom management include personalized physical therapy [11] and exercise. These strategies yield clinical benefits to early-stage OA, but their efficacy in later stages of OA is debated [12].

Pain treatment relies on analgesic drugs, the use of which is however a matter of discussion among clinicians due to the high cost-benefit *ratio* [13,14]. To date, despite advances in drug delivery systems, drugs for no resolutive therapies for OA are available, forcing end-stage patients to resort to surgery [15,16]. The most common drugs include oral and topical non-steroidal anti-inflammatory drugs (NSAIDs), shown to be the most effective, especially on pain reduction, and paracetamol and opioids, which proved to be less effective and linked to a higher risk of severe side effects. Topic and intra-articular are the most common delivery systems, but due to the occluded structure of the joint and the resulting low bioavailability, they pose a challenge in terms of efficacy. To overcome this issue, new therapeutic and delivery approaches, including microspheres, nanoparticles, and hydrogels, have been under development in the past decade, but most systems still present limitations [13,14,15,16,17,18,19].

Although constant progress is being made in OA research [20], the precise mechanisms underlying its pathogenesis have not yet been fully elucidated.

In the past, osteoarthritis was simplistically interpreted as the result of the stressful action of biomechanical factors leading to the “wear and tear” of the articular cartilage. However, the significant role of inflammation in both the onset and progression of OA is now well recognized. Indeed, an inflammatory state can be detected in OA patients both locally and systemically. This suggested an important role of inflammation, which however shows a lower intensity in patients with OA compared to patients with rheumatoid arthritis (RA), who serve as controls [21].

Several studies correlate the onset of OA with the establishment of a sustained condition of low-grade systemic inflammation, in turn resulting from the loss of cellular homeostasis [22,23]. This age-related phenomenon has been termed “inflammaging” [24], and its hallmarks appear to manifest in OA.

With advancing age, cellular homeostasis in chondrocytes, the only cell type present in articular cartilage, is progressively lost, and a peculiar pathological phenotype, characterized by nine hallmarks, takes over normal cellular functions [25,26]. Another surpassed misconception regarding OA is that only articular cartilage is involved in its etiopathogenesis. In fact, OA is now referred to as a “whole-joint disease” in which each compartment (articular cartilage, synovium, subchondral bone, infrapatellar fat pad, ligaments, and menisci) contributes to its progression [27]. Regarding ligaments and menisci, their involvement is particularly associated with traumas occurring at a young age, which act as a pivotal risk factor to later develop post-traumatic OA, another subtype of the disease [28]. Of notice is also the key role played by the subchondral bone. This compartment is connected to articular cartilage at the level of the tidemark, the zone where endochondral ossification takes place. Current evidence suggests that the subchondral bone is mainly involved in late-stage OA, contributing to pain and innervations and vascularization of the damaged articular cartilage while perpetrating damage by releasing catabolic factors. Nevertheless, the subchondral bone is also subject to microdamages, detectable at the radiological level, resulting from an excess of repetitive loading, which could contribute to OA in earlier stages [27,29,30,31,32]. Furthermore, Hoffa’s fat pad, the infrapatellar fat pad included within the synovial cavity, is responsible for the sustainment of low-grade inflammation through the release of adipokines and chemokines following synovitis and cartilage damage. Hoffa’s fat pad also seems to have a role in pain perception and is arising interest for potential development of cellular therapies based on resident stem cells present in the adipose tissue [33,34].

Despite the increasing knowledge regarding the involvement of different compartments in OA pathogenesis, the spark originating the disease is still greatly debated. In this regard, in recent years, the synovium, due to its pivotal role in triggering the immune response in OA, has turned from an extra to a lead actor.

## 2. Synovitis in Osteoarthritis

The synovium, or synovial membrane, consists of a connective tissue surrounding the inner surface of diarthrodial joints such as the knee and elbow. The synovium is made up of two layers: the outer layer, or *subintima*, which consists of a loose connective tissue that holds blood vessels, lymphatic vessels, and nerves, and an inner layer, or *intima*, where the synovial cells, called synoviocytes, reside. These cells are in turn classified as type A (macrophage-derived) and type B (fibroblast-derived) depending on the progenitor.

In different articular diseases, synovial tissue is subject to inflammation-driven changes leading to inflammation of the synovium or synovitis. Morphological changes in the synovium include increased cellularity at the lining and sub-lining levels, tissue hyperplasia, neoformation of blood vessels and afferent nerve fibers, and activation of the immune system. This last feature, primarily, is characterized by infiltration of immunocompetent cells recalled from the bloodstream [22,35,36,37,38].

Although synovitis has been predominantly associated with rheumatoid arthritis (RA), it is estimated that most OA patients develop synovitis [39]. Evidence collected through imaging and histopathologic analyses has demonstrated that synovitis facilitates the pathogenesis of OA [40].

In fact, current knowledge of the mechanisms leading to the insurgence of synovitis in OA refers to the activation of innate immunity. Following the loss of homeostasis in chondrocytes, the release of proinflammatory mediators leads to a progressive degradation of the extracellular matrix (ECM). Consequently, ECM fragments are released into the synovial cavity, where they activate native immune cells (mostly consisting of macrophages) and synovial fibroblasts. These cells are in turn responsible for the production and release of other inflammatory mediators, including cytokines, chemokines, lipid mediators, and, most importantly, alarmins.

## 3. Innate Immunity in Osteoarthritis

Innate immunity is the first line of defense activated by vascularized tissues when they suffer damage. Although the relevance of adaptive immunity in synovitis, particularly in relation to T-cells, has been widely demonstrated both in OA and RA [37,41,42,43], innate immunity is the first actor in the chain effect underlying synovitis involvement in OA. In OA, the combination of impaired cellular homeostasis, low-systemic grade inflammation, and deficient immune response combine into a perfect storm sustaining the progression of the disease. Indeed, unlike adaptive immunity, the innate response relies on the recognition of highly conserved molecular patterns arising from pathogens (PAMPs, pathogen-associated molecular patterns) or released by necrotic cells at the site of tissue damage known as damage (the aforementioned DAMPs) [44].

**Figure 1:**
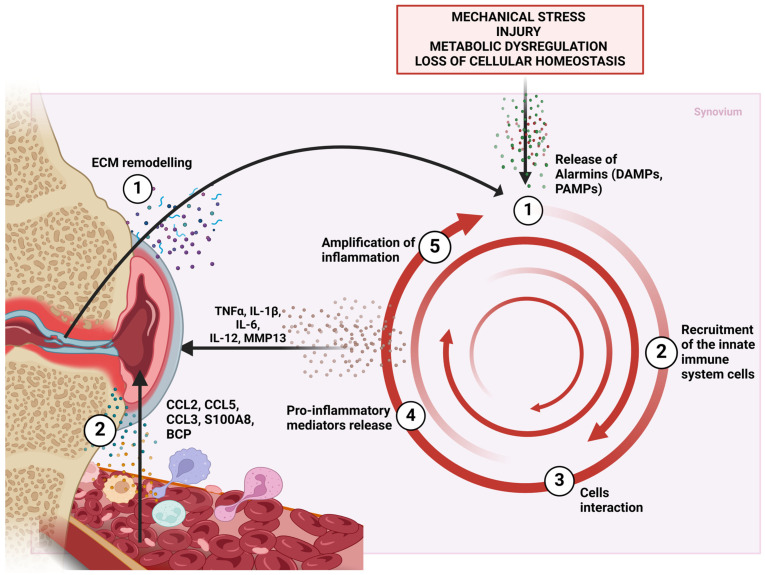
Cellular mechanisms involved in OA synovitis in osteoarthritis. OA is a multifactorial disease characterized by several risk factors such as mechanical stress, injury, metabolic dysregulation, but, above all, loss of chondrocyte cellular homeostasis that affects all other cells resident in the joint tissues. Deregulation at the cellular level triggers inflammatory mechanisms and determines the establishment of a self-sustaining low-grade inflammation, also known as “inflammaging”. The triggering of synovitis in OA is not entirely clear due to the overlap and mutual dependence of the mechanisms involved. The most accepted theory is that the loss of chondrocyte homeostasis is the first step in the chain of events. Chondrocytes are the only cell type of articular cartilage. Activation of inflammation-related signaling pathways induces the release of cartilage matrix-degrading enzymes such as MMP-13 and MMP-3, responsible for ECM remodeling in articular cartilage. ECM fragments (through the DAMPs they expose and the subsequent interaction of DAMPs with PRRs of immune cells) and proinflammatory mediators (such as cytokines) stimulate the inflammatory response of the synovium (1). Activation of inflammation leads to the recruitment of innate immune cells from the bloodstream (2) along with the activation of immunocompetent cells that reside in the synovium (mainly macrophages). The interaction between different immune cells in the synovium (3) fuels the production of multiple proinflammatory factors (4) that cause further tissue damage (5). This endless cycle is pivotal in sustaining the inflammatory *milieu* leading to the progression of the OA. Abbreviations: DAMPs, danger-associated molecular patterns; PAMPs, pathogen-associated molecular patterns; PRRs, pattern recognition receptors; CCL, C-C motif chemokine ligand; IL, interleukin; TNF-α, tumor necrosis factor-α; MMP, matrix metalloprotease.

DAMPs trigger inflammatory responses by interacting with receptors (PRRs, pattern recognition receptors) of innate immune cells. Once activated, innate immunity should trigger a response towards tissue regeneration or repair. In the case of OA, however, this activation results from improper signals derived from deregulated cells. The obtained effect is therefore opposite to the physiological one and leads to the establishment of a hostile immune response that promotes tissue damage [44]. The main aspects of how immune cells generate and maintain the low-grade inflammation of synovium that causes OA progression are recapped in Figure 1, without neglecting the role of the loss of chondrocyte homeostasis in the onset of inflammation. The different components of innate immunity and the mediators they produce crosstalk through a complex communication network. In a recent review, Kuang et al. described the possible benefits of Chinese traditional medicine in what they delineate as the “immune-joint” axis in OA [45]. To properly address the issue, first the contribution of each population has to be elucidated. Using CIBERSORT for deconvolution of global gene expression data, researchers characterized the immune cell landscape in various knee structures affected by OA [46]. This analysis revealed significant differences in the proportions of immune cells between normal and osteoarthritic tissues. In OA synovial tissues, there were notable increases in plasma cells, M1 and M2 macrophages, activated dendritic cells (DCs), and resting mast cells (MCs).

In summary, the OA progression towards the end stage is guided by the previously described changes. It is sustained by a vicious cycle that triggers the continuous release of inflammatory factors and stimulates oxidative stress, thus exacerbating the homeostatic deregulation of the various populations of cells in the joint.

In this review, we aim to summarize current knowledge on the role of innate immunity cells in synovitis and emphasize their effect in the pathophysiology of OA. Moreover, we aim to analyze different potential strategies to target innate immunity for therapeutic purposes in OA synovitis.

## 4. Materials and Methods

Studies for this review were selected using up-to-date databases, including Pubmed, Scopus, and Web of Science. The name of the cell type, together with “synovitis”, “osteoarthritis”, “innate immunity”, and “inflammation” were the keywords used in the search. Due to the paucity of studies matching our criteria, no time limit was applied in the research, except for macrophages (last five years), given the abundant scientific production on this cell type. In the selection of articles, the criterion “studies reported in English” was introduced, excluding articles written in a language other than English to overcome language barriers.

## 5. Innate Immunity Cells in Synovitis in OA

### 5.1. Myeloid-Derived Suppressor Cells

Myeloid-derived suppressor cells (MDSCs) are a group of immature immune cells of bone marrow myeloid derivation. They were initially identified in correlation with tumors, where their main function consists in shutting down the host immune response [47]. MDSCs display different phenotypes and functions. They originate from a common precursor defined as e-MDSC, then differentiate into two main types: precursors of polymorphonuclear granulocytes (G-MDSC/PMN-MDSC) are characterized by a neutrophil-like phenotype; likewise, monocyte-like MDSC (M-MDSC) have similar phenotypical features to monocytes. Compared to mouse MDSCs, human MDSCs are difficult to characterize due to the multiplicity of surface markers needed for their identification [48]. This paper provides a detailed description of the phenotypic characterization of MDSCs. To simplify their phenotyping, mature MDSCs are defined as CD33+ and CD11b+ but do not express maturation markers such as HLA-DR. So, the resulting phenotypic profile is HLA-DR–/lowCD11b+CD33+CD14+CD15– for M-MDSCs. On the other hand, PMNMDSCs express HLA-DR-CD11b+CD33midCD14-CD15+. The characterization of these subtypes with the markers CD14 and CD15 clearly refers to the cell types with which they share common phenotypical features (i.e., neutrophils and monocytes). The common precursor (e-MDSC) in turn is described as HLA-DR-CD33+Lin- (CD3-CD14-CD15-CD19-CD56-). Recently, the lectin-like oxidized LDL receptor 1 (LOX-1) was identified as a novel-specific marker for PMN-MDSC, while investigations are still ongoing to identify M-MDSC markers [49]. Although initially linked to angiogenesis and metastasis formation in cancer, MDSCs have also been linked to autoimmunity and bone remodeling in RA and OA [50]. Evidence suggests two different hypotheses regarding the role of MDSC roles in RA, with data supporting both pro- and anti-inflammatory activity of this cell population. According to Zhang et al., MDSCs exert a proinflammatory effect mediated via Th17 cell activation and Treg cell inhibition [51]. Contrarily, Ark et al. proposed the opposite mechanism of MDSCs regarding Th17/Treg cell balance [52]. This discrepancy could be attributable to the different expansion patterns of Th17 cell and Treg cell populations and the plasticity between Th17 and Treg cells. The two subgroups, M-MDSC and PMN-MDSC, could exert a suppressive and/or regulatory function exploiting different mechanisms of action [53]. On the other hand, the role of MDSCs is not clear in OA, but recent studies shed light on a possible role in the disease. First, a single-sample gene set enrichment analysis (ssGSEA), aiming to evaluate the infiltration of immune cells in OA, showed that MDSCs were deregulated in OA samples along with immature B cells, MCs, NK, and several adaptive immunity cells [54]. Another link between MDSCs and diseases characterized by bone loss or reabsorption, such as OA, lies in their capacity, being macrophage precursors, of differentiating into osteoclasts. Evidence of that comes from research on the tumor microenvironment, where MDSCs increase in number was linked to their differentiation in osteoclast progenitors leading to cancer-associated bone destruction [55,56,57,58]. This differentiation seems to be driven by an inflammatory environment. Further confirmation of this pro-catabolic activity after differentiation comes from several studies involving bone-remodeling in different disease models like periodontitis and osteoarthritis [51,59,60,61]. Zhang et al. also showed an increase of the M-MDSC number in obese subjects. This connection was confirmed in a DMM OA model in mice where a hyperlipidic diet led to an increment in MDSCs, which in turn differentiated in osteoclasts in connection with OA progression. These data suggest that MDSC differentiation can be reprogrammed in metabolic disorders and that this cell population could actively contribute to OA onset and progression [62]. Lastly, a recent study by Kwack et al. confirmed that M-MDSCs represent a distinct osteoclast precursor population, therefore validating M-MDSCs as a possible target to limit bone destruction in OA [61].

### 5.2. Monocytes

Monocytes are recognized as the largest type of leukocyte in the blood. They have the capacity to differentiate into macrophages and monocyte-derived dendritic cells, contributing significantly to the innate immune system. These cells not only participate in innate immunity but also influence adaptive immune responses and play crucial roles in tissue repair. The first clear identification of monocyte subsets using flow cytometry was documented in the late 1980s [63], revealing a population of CD16-positive monocytes [64]. Currently, human blood is known to contain three distinct types of monocytes based on their phenotypic receptors [65]. Classical monocytes (CD14++ CD16−) are characterized by a high level of CD14 cell surface receptor expression. These monocytes are the most represented in OA [66] and play an important role in its aggravation through the secretion of inflammatory cytokines [67]. Non-classical monocytes (CD14+CD16++) display low levels of CD14 and co-express the CD16 receptor. Intermediate monocytes (CD14++CD16+) express high levels of CD14 and lower levels of CD16 [65]. Monocytes are dynamically active cells and migrate from the bloodstream to sites of inflammation to perform their functions [68]. Upon migration, they can differentiate into macrophages and dendritic cells. Broadly, monocytes and their progeny serve three primary functions in the immune system: phagocytosis, antigen presentation, and cytokine production. Monocytes play a crucial role in the pathogenesis of osteoarthritis (OA) and synovitis. Each monocyte subpopulation can also carry out specific functions independently. By mediating the activation inflammatory response, monocytes could contribute significantly to the progression of pathological conditions such as OA. In fact, in addition to the self-maintained lining and interstitial sub-lining macrophages present under normal conditions, the arthritic synovium also includes inflammatory monocyte-derived macrophages [69]. Monocytes are recruited to the joint by chemokines via the CCL2/CCR2 and CCL5/CCR5 axes. Specifically, the CCL2/CCR2 axis is a critical mediator of monocyte recruitment, inflammation, and cartilage destruction. The inhibition of this pathway in mouse models significantly reduces OA severity [70]. Mondadori et al. developed an organotypic microfluidic model to study monocyte extravasation into the synovium, showing that blockade of CCR2 and CCR5 receptors effectively inhibited monocyte extravasation [71]. Recently, Zhao et al. suggested that the CCL3 produced in osteoarthritic knees of a mouse model can attract circulating monocytes to the inflamed synovium through the CCR1 receptor [72]. Moreover, CX3CL1/CX3CR1 signaling also plays a role in recruiting monocytes, with elevated levels of CX3CL1 detected in the peripheral blood [73] and synovial fluid [74] of OA patients. According to this evidence, monocytes are recruited by different chemokines released by distinct cell subtypes in response to various inflammatory stimuli. The study of these processes could lead to therapeutic prospects [75]. A meta-analysis performed by Ni et al. has demonstrated elevated levels of monocyte chemotactic protein-1 (MCP-1 or CCL2) in OA patients compared to healthy controls [76]. Women with knee OA exhibit higher levels of monocyte activation markers like CD16, CCR2, and HLA-DR. This activation is associated with higher serum TNF levels, BMI, and greater pain severity, suggesting a link between systemic inflammation and OA symptoms [77].

Once recruited to the joint, monocytes differentiate into macrophages under the influence of the local microenvironment. These macrophages can adopt different phenotypes, broadly categorized into pro-inflammatory (M1) and anti-inflammatory (M2) macrophages [78,79]. Most studies on OA joints have identified inflammatory monocyte-derived macrophages as the primary agents responsible for promoting and sustaining inflammation [80,81].

All these processes facilitate the establishment of an inflammatory cycle in which the release of pro-inflammatory cytokines by monocytes induces the recruitment of other immune cells that in turn release additional pro-inflammatory mediators, perpetuating articular damage.

In addition, activation of monocytes and release of their products not only affect cartilage and synovium but also promote osteoclastogenesis and bone resorption [82]. Hirose et al. proposed that CCL2 secreted by osteoblasts activates the monocytes to form mature osteoclasts, potentially explaining the accelerated osteoclastogenesis and joint destruction observed in inflammatory conditions [83]. Furthermore, the role of monocytes and their activation and release of pro-inflammatory molecules in the synovium has been correlated with the Kellgren and Lawrence scale [84] and the severity of symptoms, specifically stiffness, function, and quality of life [85]. Given the importance of monocytes in the pathogenesis and progression of OA, these cells are now being investigated as a prognostic biomarker in OA. In this regard, supporting evidence has emerged. In fact, the neutrophil-to-monocyte ratio is independently and inversely correlated with OA severity, as determined by the Kellgren–Lawrence scale [86]. while the monocyte-to-lymphocyte ratio is a reliable predictor of OA progression [87].

### 5.3. Macrophages

Macrophages are the most important component of the innate immune system and exert a pivotal role as first-line defense not only against pathogens but also in the tumor microenvironment [88]. Widely studied for their essential function in maintaining immunity homeostasis in health and disease, macrophages also play a crucial role in the onset and progression of synovitis and therefore OA. Since a detailed state of the art regarding this cellular subtype in OA has been reviewed in recent monothematic papers [89], we will limit our analysis solely to functional aspects of macrophages in synovitis associated with OA.

Macrophages derive from the maturation, in tissues, of monocytes migrating from the circulation. Upon maturation, macrophages acquire a specific phenotype depending on the functions they perform. Based on these criteria, macrophages can be classified into three types: M0, M1, and M2. Under specific inflammatory stimuli, M0 can polarize into M1 and M2 phenotypes [90]. On one hand, M1 are commonly described as proinflammatory cells with anti-pathogen functions. Polarization towards the M1 phenotype can be stimulated by lipopolysaccharide (LPS), the major component of the outer membrane of Gram-negative bacteria, and cytokines, such as IFN-γ and TNF-α. M1 macrophages can in turn secrete pro-inflammatory cytokines/chemokines, such as IL-β. M2 macrophages, on the other hand, are recognized to exert anti-inflammatory and tissue repair/regenerative functions. They are induced by IL-4 and IL-13 [91]. In the normal synovial membrane, type A synoviocytes, which are macrophage-like cells, are physiologically present in the lining layer, and their number increases in parallel with the progression of OA and is accompanied by the appearance of a low-grade synovitis [37]. Zhang et al. demonstrated that the increase in M1 macrophages, compared to M2, leads to a worsening of OA in mice via R-spondin-2 (Rspo2), a protein they can secrete [92].

Macrophages are the most represented immune cell population in the synovial infiltrate of patients with OA, amounting approximately to 65% of the total and followed by T cells, which make up for about 22% of the infiltrate [93]. Unlike what happens in inflammatory synovitis associated with rheumatoid arthritis, infiltration of immunocompetent cells in OA synovitis is not as diffuse; rather, it is limited to the sites of chondropathy. The increase in the number of macrophages and their M1/M2 polarization is, however, the result of interactions with other cells of the joint (chondrocytes, synoviocytes, and lymphocytes). This complex network of cells releasing molecules to attract macrophages and other immunocompetent cells on site contributes to igniting and perpetuating synovitis in OA. In this scenario, molecules such as soluble matrix degradation products (SMDPs) from cartilage, fibronectin, and adipokines are the main players. Also, DAMPs, by interacting with PRRs (such as Toll-like receptors, TLRs), induce macrophages to produce inflammatory cytokines and chemokines. Finally, growth factors, interleukins, and chemokines, particularly the monocyte chemotactic protein-1 (MCP-1), are the last effectors that via specific receptors activate the JAK-STAT signaling pathway [94]. The importance of macrophages in producing mediators concurring to the progression of OA has been clarified by Van Lent et al., who demonstrated that a depletion of synovial macrophages is associated with a decrease in the production of bone morphogenetic proteins BMP-2 and -4 and thus also in osteophyte formation [95]. Similar results were confirmed in other independent studies [96,97]. Furthermore, Takano et al. found a correlation with a decrease in nerve growth factor (NGF) expression, a pain-related cytokine [98]. In this regard, as early as 2016, Kraus et al., using radionuclide-labeled folate (etarfolatide), provided direct in vivo evidence linking the amount of activated synovial macrophages to pain in OA [99]. The mechanisms that lead M1 macrophages to trigger inflammation at the joint level arise from the increased expression of proinflammatory factors such as IL-1β and IL-6, MMP13 and ADAMTS5, and the suppression of aggrecan and collagen II. In turn, M2 (induced by IL-10) promoted the expression of IL-1β and SOCS1-suppressor of cytokine signaling 1 (SOCS1) [100]. Another important function of synovial macrophages in innate immunity was proposed by Culemann et al., who identified a type of tissue-resident macrophages with a CX3CR1+ phenotype capable of forming a dynamic membrane-like immunological barrier at the synovial lining that armors the joint cavity [101]. The altered activity of macrophages was also reported as a contributor to chronic inflammation of OA [102]. In agreement with this hypothesis, Liu et al. showed, in both preclinical and clinical studies, that obesity induces the switch from the M2 to the M1 phenotype [103]. In addition, the synovial fluid of patients with OA exhibits an increased ratio of M1/M2 macrophages in correlation with OA severity [103]. Macrophages also participate in homeostasis maintenance phenomena such as that of efferocytosis, which regulates the turnover of apoptotic cells and bone resorption/remodeling. The impairment of these mechanisms could contribute to OA progression [104]. Very recently, Luo et al. have well reviewed the whole network of chemokines and cytokines produced by monocytes and macrophages in OA pathogenesis. Several monocyte/macrophage chemokines, by interacting with their receptors on target cells, can act as pro-inflammatory mediators in OA. Among these are the following: the macrophage inflammatory proteins (MIP)-1α/CCL3, MIP-1β/CCL4, and MIP-3α/CCL20; the monocyte chemotaxis proteins MCP-1/CCL2 and MCP2/CCL8; the chemokine CCL17, also known as thymus and activation-regulated chemokine (TARC); the macrophage-derived chemokine CCL22; and RANTES (RANTES is the acronym for regulated upon activation, normal T cell expressed and secreted; RANTES is also called CCL5) [105].

### 5.4. Dendritic Cells

Dendritic cells (DCs) are pivotal in the immunopathogenesis of synovitis and therefore OA. These specialized cells bridge the innate and adaptive immune systems, influencing both local and systemic inflammatory processes. Their diverse roles encompass antigen presentation, cytokine production, and the modulation of inflammatory responses [106]. Recent investigations have elucidated the multifaceted contributions of DCs to OA pathogenesis. By interacting with various immune mediators and cell populations, DCs regulate complex mechanisms underlying joint inflammation and tissue damage. DCs are a heterogeneous group of cells, characterized by their differential expression of essential transcription factors like interferon regulatory factor 4 and 8. This diversity allows the identification of two different types of dendritic cells: conventional/myeloid DCs (cDCs) and plasmacytoid DCs (pDCs) [106,107]. Myeloid cDC1s are known to promote T helper type 1 (Th1) and natural killer (NK) responses through the production of -IL-12 and activation of CD8+ T cells via MHC class I. On the other hand, cDC2s produce higher quantities of IL-12 and are potent activators of Th1, Th2, Th17, and CD8+ T cells [107,108]. DCs play a pivotal role in promoting Th17 cell differentiation by stimulating memory CD4+ T cells to produce IL-17. This mechanism contributes to the maintenance of inflammatory processes in OA and synovitis, highlighting the intricate interplay between DCs and adaptive immune responses [109]. Another subset of monocyte-derived DCs, called inflammatory DCs, are typically present in inflammatory conditions, including synovitis, psoriasis, and inflammatory bowel disease [107,108,110]. Marzaioli et al. have identified a novel DC population characterized as CD209/CD14+ DCs in inflammatory arthritis. These cells are characterized by classical DC markers (HLADR, CD11c) and, in addition, the monocytic marker CD14. These DC subtypes express higher levels of cytokines like IL-1β, IL-6, IL-12, and TNFα and exhibit unique chemokine receptor profiles compared to healthy controls, contributing to their inflammatory and migratory capacities. This evidence opens new avenues for future therapeutic interventions targeting these specific DC populations, particularly through the JAK/STAT signaling pathway, which regulates their activation [110]. In another study conducted by Xiaoqiang et al., DC number resulted in increased in the synovium of early-stage OA rabbits (2 to 4 weeks post establishment of surgical OA model). At the same time, they noticed that an increment in DCs number was associated with an increase in IL-lβ and TNF-α expression and a high degree of synovial inflammation. Interestingly, inflammation of the synovium decreased along with DCs number. These results suggest a possible role of DCs in early-stage OA onset of inflammation in the synovium [111]. Another mechanism of action of DCs was assessed in an iodoacetate OA model aiming to investigate the role of TLRs in DCs-mediated inflammation. Evidence shows a markedly increased expression of TLR family members in DCs derived from the synovial fluid of OA mice compared to controls. Moreover, the exposition to TLR agonists such as LPS and R848, led to massive DCs-mediated release of inflammatory cytokines, although their maturation status remains unchanged. The use of a TLR antagonist, FP7, inhibited cytokine production and attenuated the overall inflammatory response in OA, both in vitro and in vivo [112]. Further confirmation of the crucial role of TLRs in the activation of DCs in OA comes from Segura et al., who showed that TLR4 is involved in obesity-induced OA in mouse models [113]. In addition, in the same model they demonstrated the correlation between the increased expression of TLR in DCs with the number and inflammatory activity in the adipose tissue of obese non-diabetic humans compared to controls. In this environment, DCs contribute to inflammation by promoting the switch towards Th17 cells, commonly associated with insulin resistance in obesity [113]. This connection between metabolic status and immune activation further complicates the inflammatory *milieu* in OA, especially of TLR4 in obesity-induced OA, suggesting that metabolic deregulation intensifies DCs-mediated inflammation in OA [113,114]. Moreover, DCs facilitate important molecular interactions between synovial cells and chondrocytes in OA. Chou et al. showed that cytokines were produced and then released to the joint space by HLA-DRA+ synoviocytes (macrophages and DCs), but not chondrocytes [115]. This *milieu* not only promotes synovial inflammation but also contributes to cartilage degradation, suggesting that DCs within the synovium significantly contribute to OA pathogenesis. The strategic targeting of DCs and their cytokine production may thus offer novel therapeutic opportunities to manage OA progression. As previously described, tissue changes in the inflamed synovium are sustained by alarmins, such as S100 proteins, the high mobility group protein B1 (HMGB1), and IL-33, released during cell stress and necrosis. Alarmins recruit DCs to inflamed synovial tissues and are responsible for initiating and perpetuating the adaptive immune response [116]. These mediators bind to specific receptors on DCs, promoting their maturation and the subsequent polarization of T cells into pro-inflammatory subsets, sustaining chronic inflammation and joint damage. Exposure to basic calcium phosphate crystals elicits robust activation of primary human macrophages and dendritic cells in OA, triggering signaling pathways involving Syk and PI3K leading to the production of pro-inflammatory cytokines and damage-associated molecules, exacerbating joint inflammation [117]. In conclusion, dendritic cells contribute to the immune response in synovitis, influencing both the initiation and progression of OA. Their ability to link adaptive and innate immunity, respond to TLR agonists, and induce pro-inflammatory cytokine production also makes them key players in the inflammatory crosstalk.

### 5.5. Neutrophils

Neutrophils (NTs) are the most abundant leukocytes in the body, constituting 40–60% of their totality in the bloodstream and representing an indispensable component involved in all aspects of the innate immune system. NTs are quickly mobilized to inflamed areas in response to cytokines and chemokines, where they adhere to endothelial cells. During inflammation and infection, NTs have the capability to produce and secrete different types of molecules, which serve to eliminate pathogens through phagocytosis, chemical degranulation, and the formation of the so-called neutrophil extracellular traps [118]. Although mostly implicated in defensive responses, NTs also contribute to the inflammatory response in several degenerative diseases and play a pivotal role in tissue damage in arthritis. Despite being predominant in the infiltrate in the synovium in RA, NTs are also among the first immune cells to infiltrate the synovium in OA [119]. Accounting for 26% of total cells, they represent the most abundant immunity cell population in the synovium, second only to macrophages. The degranulation, production of reactive oxygen species (ROS), and delayed apoptosis of NTs correlate with the severity of synovial inflammation and the destructive potential of joint tissue, suggesting a synergistic activity of granule mediators and ROS in articular cartilage degradation [120]. In a recent study, Hsueh et al. compared NTs to macrophages in synovial fluid (SF) to identify possible predictive biomarkers of OA. This study showed that while macrophages were linked with transforming growth factor beta-1 (TGF-β1), NTs were strongly associated with elastase, and both molecules correlated with radiographic KOA severity. Moreover, baseline synovial fluid concentrations, in conjunction with radiographic scores, predicted KOA progression [121]. In another study conducted by Haraden et al., six different biomarkers of ECM degradation (MMP-3, TIMP-1), angiogenesis (VEGF), vascular adhesion (sVCAM-1, sICAM-1), and inflammation (MCP-1) were associated with both NTs and macrophages and correlated to the grading of synovitis [122]. These results suggest that NTs mediators could serve as useful biomarkers for disease progression. In fact, neutrophil elastase is a well-known proteoglycan-degrading enzyme historically associated with inflammatory arthritis, yet recent evidence hints at its potential implications in OA. Wilkinson et al. investigated the impact of NE on the destruction of collagen in OA cartilage and the activation of collagenase in ex vivo and in vitro human OA cartilage [123]. Their results show that NE directly and vigorously activated pro-MMP-13, leading to the generation of the fully active form. Treatment with sivelestat, an inhibitor of NE, significantly reduced the pathological processes in OA model rats in vivo [124]. Also, KOA NTs appear to express a specific profile and phenotype compared to infection (INF)-NTs. KOA NTs showed lower expression levels of CD11b, CD54, and CD64, along with an increased expression of CD62L, TLR2, and TLR4 compared to INF-NTs. Additionally, KOA SF, as expected, showed lower levels of inflammatory mediators and proteases compared to INF-SF, except for CCL2. Functionally, KOA NTs displayed an increased production of ROS and enhanced phagocytic activity compared to INF-NTs. Furthermore, KOA and INF-NTs differed in cell sizes, histological characteristics of surrounding synovial tissues, and their effects on human umbilical vein endothelial cells [125]. The activation of NTs also contributes significantly to the release of various proinflammatory cytokines (IL-1, IL-6, IL-21, IL-22, IL-23,TNF-α,TGF-β) in the SF, thus influencing OA progression [126]. Certainly, their presence highlights the intricate balance between pro- and anti-inflammatory activities, contributing to the characteristic low-grade inflammation observed in synovitis and OA. The perpetration of this vicious cycle sustained by NTs among other cells in the synovium is responsible for narrowing the possibility of treatments for OA [127]. NTs also release powerful mediators in the synovium that modulate the activity of other cells of the immune system. Benigni et al. demonstrated in in vivo depletion experiments that NTs contribute to an increase in NKs within the synovium by expressing CXCL10 in inflamed joints. Hence, NTs and NK cells serve as crucial immune cells promoting OA progression, with their functional interaction facilitated by the CXCL10/CXCR3 axis [128].

### 5.6. Eosinophils

Eosinophils are a small subset of circulating granulocytes, accounting for 2–4% of the total white blood cells [129]. Traditionally, they have been identified as end-stage effector cells, primarily involved in defending the host against helminth infections. However, their role in promoting allergic responses is usually held in greater consideration [130]. In fact, they trigger the onset of symptoms associated with allergic reactions by releasing inflammatory mediators that increase vascular permeability and attract other immunity components to the site of inflammation [131]. Studies regarding the role of eosinophils in the pathogenesis and progression of osteoarticular diseases are few, but recently the focus on this cell type has increased due to their putative protective role in OA. Indeed, a recent study suggests that eosinophils, as a crucial component of T helper 2 (Th2) immune responses, exert an anti-inflammatory effect in arthritis and RA [132]. Notably, activated Th2 and type 2 Innate lymphoid cells (ILC2) cells in cartilage, in response to proinflammatory stimuli, release IL-5 and recruit eosinophils, which in turn induce the release of IL-4 and IL-13 [132,133,134]. The regulation of these mediators could be relevant to treat synovitis and is supported by a significant amount of experimental data.

Chen et al. showed that the IL-4/IL-13-induced STAT6 signaling pathway results in the build-up of eosinophils within the joints, leading to significant suppression of arthritis and safeguard against bone loss [135]. The role of IL-4 treatment in slowing down disease progression has also been confirmed in animal models of experimental arthritis [136,137]. Furthermore, arthritis severity increased significantly in IL-4Rα knockout mice [138]. In mice, treatment with IL-13 reduced both the severity and incidence of arthritis, demonstrating anti-inflammatory properties [139] previously shown in macrophages [68,70,71]. Another mechanism underlying the activity of eosinophils appears to be the release of anti-inflammatory lipids, such as protectin D1, which exhibited tissue-protective properties in both acute peritonitis [140] and a mouse model of arthritis [141].

Given the dual nature of eosinophils as pro-inflammatory and pro-resolutive cells, it is plausible to assume the existence of different subpopulations with distinct biological functions. Particularly, a recent study identified a subgroup of *regulatory eosinophils* (rEos) in the joints of arthritic mice and in the synovium of patients with RA remission. rEos release anti-inflammatory molecules, promoting tissue regeneration contrary to classic eosinophils found in allergic lung diseases [142]. Overall, data from experimental studies have correlated the quantity and activity of eosinophils to bone mass in healthy individuals and those with rheumatoid arthritis, suggesting that eosinophils play a regulatory role in musculoskeletal health [143]. Recent scientific evidence demonstrates a possible protective role of eosinophils in the resolution of osteoarticular diseases.

As already reported for other cell types, the number of eosinophils in synovial tissues of OA patients is significantly increased compared to healthy controls [139]. Furthermore, a biomarker expression study of synovial immune cell infiltrates of OA patients identified a significant correlation between pyruvate dehydrogenase kinase 1 receptor (PDK1) expression and eosinophils [144]. Moussa et al. were the first to show the increase in eosinophil number in the subcutaneous adipose tissue of patients with metabolic syndrome, suggesting an active role of eosinophils in establishing its characteristic inflammatory state [145]. Given the increase of metabolic OA in the population, further studies are needed to elucidate the role of eosinophils in synovitis and possible therapeutic use.

### 5.7. Basophils

Compared to other cell types, very little data from the literature focus on the link between basophil granulocytes and OA synovitis. In a very recent genomic analysis, Liu et al. highlighted a difference in the gene expression of the basophil infiltrate of OA patients compared to control, suggesting a possible involvement in the pathogenesis [146].

Noteworthy, basophils express the CD40 receptor, which, following activation through its ligand (CD40L), induces the proliferation of synovial fibroblasts and induces them to produce IL-6, contributing to inflammation in synovitis [147]. Although not much is known about the role of basophils in OA, they appear to be implicated in autoimmune conditions of the joints, like RA [148], but the underlying mechanisms by which they act are yet unknown.

### 5.8. Mast Cells

Mast cells (MCs) are cells of myeloid derivation, present in a small number in most of the connective tissues of the body. Although predominantly linked to type II immune responses following microbial infections and allergic reactions, MCs are a versatile cell type involved in the regulation of both physiological and pathological mechanisms. Indeed, their contribution is essential for angiogenesis regulation, vasodilation, and vascular permeability. MCs are on the front lines of defense against pathogens [149]. Furthermore, they are involved in inflammatory diseases affecting multiple organs, such as the lungs, intestine, adipose tissue, and articular joints [150].

An explanation for the pleiotropic nature of MCs could reside in their ability to secrete numerous soluble mediators of inflammation, such as histamine, heparin, proteases, and cytokines. These molecules are normally stored in intracellular granules and release only in response to an external inflammatory stimulus. The activation of these mediators is responsible for the activation or inhibition of other immune cells but also of endothelial cells, fibroblasts, and other somatic cells that sustain the inflammatory response [151].

MCs can also synthesize *ex novo* lipidic mediators like prostaglandins and leukotrienes, which intervene in the regulation of ongoing inflammatory responses. The response of MCs is triggered via the IgE receptor FcεRI or the complement receptor C5aR and induces degranulation followed by *de novo* synthesis and release of other soluble mediators. Another possible mechanism passes through the activation of the TLRs but, in this case, without inducing degranulation [152,153]. Moreover, tryptase, a serin-protease released from MCs, can activate the protease-activated receptor 2 (PAR-2), triggering a pro-inflammatory signaling response [154].

In synovial tissue, MCs develop following the maturation of hematopoietic precursors, which are induced to differentiate by stem cell factors and other growth factors secreted by local cells resident in synovium. Once mature, MC cells range between 1 and 5% of all hematopoietic cells in the human synovium. Interestingly, MCs are detectable in the synovium of healthy individuals, suggesting a role of these cell in tissue homeostasis, but an increase in their number has been shown in association with both RA and OA [155,156]. Indeed, the presence of MCs has been associated with the activation of inflammatory and degenerative mechanisms sustaining the progression of these pathological conditions. Evidence suggests that, concurrently with the establishment of the inflammatory state in RA and OA, MCs accumulate in the synovium, where they are activated by inflammatory factors. Following, their degranulation and release of mediators is responsible for the acute and chronic phases of articular inflammation. During acute inflammation, mediators released by MC degranulation are responsible for the so-called “jump start” signal, which triggers the vascular phenomena of acute inflammation (angiophlogosis). Other MC mediators interact with macrophages, histiocytes, and fibroblasts, triggering a ripple effect that sustains joint-damaging, low-grade inflammation [157]. MCs are the most numerous immune cell type in OA, together with macrophages and T lymphocytes. Furthermore, MCs are present in greater numbers in OA synovium rather than RA, suggesting a key role in its pathogenesis. However, the mechanisms underlying their contribution to the onset and progression of OA have not yet been clarified, due to contradictory data from animal models and clinical studies in patients [156]. De Lange-Brokaar et al. showed that the number of MCs in OA correlates with the grade of synovitis (Krenn score) and the radiological score (Kellegren–Lawrence) [158]. Furthermore, they identified two subtypes of MCs: MCt, characterized by the presence of tryptase granules, and MCt, with granules containing both tryptase and chymase, a chymotrypsin-type serine protease. The first subtype is prevalent in OA compared to controls, and the *ratio* between ct/t is smaller compared to that of RA [158]. Moreover, Uchida et al. showed that MC markers (CD117, CD203c, and FGF2) were highly expressed in the synovium of obese patients with KOA, posing an interesting question on the role of MCs in the etiopathogenesis of metabolic OA [159]. MCs could also play a role in regulating the hyperalgesia that characterizes pain perception in late-stage OA. Indeed, evidence has been shown that MC activation increases pain in OA [160]. In their in vivo study, Sousa-Valente et al. used a common pain model of OA obtained via monoiodoacetate injection [161] in P782STrkA KI mice, carrying a mutation in the Tropomyosin receptor kinase A (TrkA), a high affinity catalytic receptor for NGF. This mutation resulted in an increase in mechanical hypersensitivity and hyperactivation of neurons. TrKA KI mice showed an increase in MCs in proximity of nociceptive fibers, responsible for pain perception. Possibly, the MCs-induced release of prostaglandin D2 (PGD2) could act as an effector of pain perception by activating nociceptors [160]. In conclusion, MCs participate in the exacerbation of low-grade inflammation of the synovium in KOA and may also play a pivotal role in pain perception.

### 5.9. Natural Killer Cells

Natural killer cells (NKs) are among the most important cells of innate immunity, known to be implied in antitumor and antiviral responses [162,163]. They are a heterogeneous population comprising several subtypes characterized by different markers, which are used to detect them. The broadest phenotype consists of CD3-CD56+ lymphocytes, which can be further classified in CD56bright and CD56dim cells. CD56bright cells are considered an immature form [164,165]. Furthermore, the various NK subtypes can differentially express a series of receptors and ligands capable of regulating the functions of other cells. The main ones are activating killer immunoglobulin-like receptors (KIRs), natural cytotoxicity receptors (NCRs [NKp30, NKp44, and NKp46]), NKG2C, NKG2D, FCγRIIIA (CD16), 2B4 (CD244), and DNAX accessory molecule-1 (DNAM-1) [166], while the main inhibitory receptors expressed by NKs cells are KIR, NKG2A, and LILRB-1 [166]. Furthermore, NKs are implicated in apoptosis. NKs can kill target cells via death ligands, such as Fas ligand (FasL) and TNF-related apoptosis-inducing ligand (TRAIL) [167,168,169]. Generally, immature forms of NKs are predominant in peripheral blood, and, once mature, they tend to downregulate CD56, upregulate CD16, and express CD57 and high levels of KIRs, mediate cytotoxic activity by secreting granzyme (Gzm) and perforin, and are described as a cytotoxic subpopulation [170]. Furthermore, the presence of memory-like and tissue-resident NK cells was reported in previous studies [171,172,173]. However, recent evidence seems to indicate that they also play a crucial role in the onset and progression of OA. Huss et al. found that the number of NKs is higher than in normal controls and that NKs are among the most numerous cells in the inflammatory infiltration of synovial tissue in OA patients [162]. In OA patients, NKs not only increase in number but also take on different characteristics. Noticeably, in OA, the percentage of CD56brightCD16 NK cells increases, and their number correlates with the severity of symptoms. Furthermore, these cells in OA patients have lower expressions of Gzm and perforin and higher expressions of IFN-γ, IL-6, TNF-α, and granulysin (known to increase inflammation). Therefore, NKs are likely co-responsible for the low-grade inflammation of OA-associated synovitis [163,174,175,176]. Specifically, in the inflammatory infiltrate of OA-associated synovitis, the most numerous NKs are CD56+CD3. These cells also seem to lose their cytotoxic characteristics and show low expression of perforin, Gzm, and KIRs and instead express CD27 and CD94/NKG2A. Synovial NKs also express CCR5 and CCR3 receptors, which, stimulated by their respective ligands present in the synovial fluid, could recruit other NKs into the joint, amplifying the inflammatory response. Huss et al. showed that the number of NKs in synovial tissue is different at different stages of OA and could correlate with disease progression [162,177]. Then NKs, through a complicated network of mediators and interfacing with synovial fibroblasts, play a regulatory role in inflammation and subsequent subchondral bone resorption. In fact, synovial NKs can switch on these processes by amplifying the RANK-RANKL axis in SF [178]. Indirectly, they also stimulate synovial secretion of IL-6 and TNF-α [179]. Conversely, SF in OA patients may reduce the production of IFN-γ and TNF-α, attenuating the pro-inflammatory effects exerted by NKs via the RANK-RANKL axis. Finally, SFs can secrete CXCL8, which attracts and activates NKs [178]. This interaction appears to be an important control system for OA-associated synovitis. Synovial NKs also have the task of eliminating senescent SFs by degranulation. This is triggered by the interaction of NKG2D with its ligand [180]. NKs are also linked to pain perception. Noticeably, the percentage of NK cells expressing Mu opioid receptors was lower in blood samples from OA patients than in pain-free patients [181].

### 5.10. A Final Glance at Innate Immunity Cells and Synovitis

**Figure 2:**
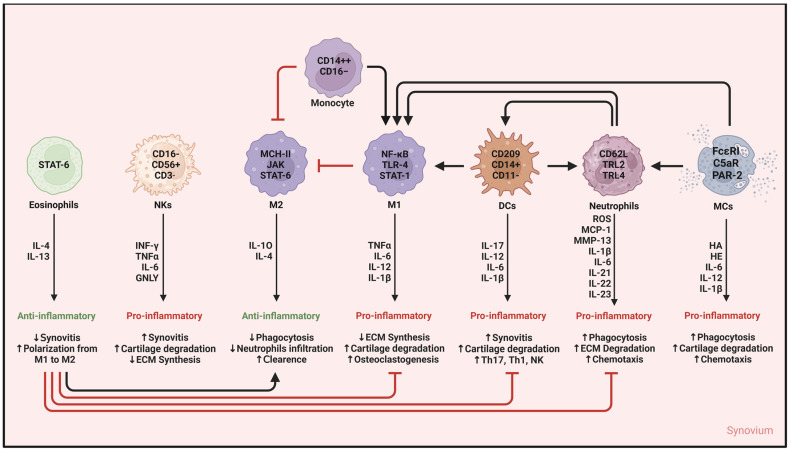
Cells of innate immunity and network of mediators in OA synovitis. Synovitis in osteoarthritis is mainly driven by the activation of innate immunity. This activation is triggered by proinflammatory factors released following the loss of cellular homeostasis. Each type of immunocompetent cell releases mediators to promote/counteract low-grade inflammation. Eosinophils and M2 macrophages exert an anti-inflammatory activity under the modulation of the JAK/STAT signaling pathway and the release of anti-inflammatory cytokines (IL-4, IL-10, IL-13). Thus, boosting these immune cells and the release of their mediators could be useful to re-establish cellular homeostasis and counteract synovitis. In contrast, M1 macrophages, NK cells, DCs, neutrophils, and MCs actively fuel the vicious cycle that keeps low-grade inflammation and synovitis alive, leading to OA progression. The release of proinflammatory mediators (such as IL-1, TNF-α, and ROS) following immune cell activation leads to worsening of synovitis features, cartilage degradation, and bone remodeling. Furthermore, the chemotactic activity of these molecules attracts more immunocompetent cells from the bloodstream, thus corroborating the proinflammatory *milieu*. Abbreviations: IL, interleukin; CD, cluster of differentiation; NKs, natural killer cells; M2, anti-inflammatory macrophages; M1, proinflammatory macrophages; DCs, dendritic cells; MCs, mast cells; Th, T helper; JAK/STAT, Janus kinase/signal transducer and activator of transcription; MHC, major histocompatibility complex; NF-κB, Nuclear factor kappa-light-chain-enhancer of activated B cells; TLR-2/4, Toll-like receptor 2/4; GNLY, Granulysin; PAR-2, protease-activated receptor 2; FcεRI, high-affinity IgE receptor; C5aR, complement receptor C5a; ROS, reactive oxygen species; MCP-1, Monocyte Chemotactic Protein 1; MMP-13, metalloprotease 13; ECM, extracellular matrix.

For a concluding synthetic overview, Figure 2 surveys all the different types of innate immune cells mentioned throughout the text and the corresponding spectrum of mediators they release in OA synovitis, recapping key information.

## 6. Conclusions

Synovitis is an inflammatory state of synovial tissue contributing to OA progression through the induction of multiple pathogenic mechanisms.

The present review summarized the substantial role played by innate immune cells in triggering and sustaining the low-grade inflammation and synovitis that insidiously fuels OA over time.

We collected important literature data supporting the key role of proinflammatory mediators released in the synovium by immune cells in OA progression and pain sensitization.

While we collected numerous studies on macrophages, we found a smaller number of studies on other cell types, such as that of basophils. Nonetheless, the evidence that has emerged encourages further investigation.

Intriguing counterintuitive findings are emerging regarding eosinophils (or perhaps eosinophil subpopulations) and their putative pro-resolving anti-inflammatory role in osteoarticular diseases.

More work is required to unravel and conceptually connect all the molecular mechanisms through which the synovial innate immune response mediates OA pathogenesis and impacts joint pain, the main symptom of OA.

The evidence available so far and discussed in this review highlights the chance for a switch in the conceptualization of OA treatment from regenerative to immune response-based approaches.

Given the critical role of immunity, targeting synovitis by fine-tuning innate immunity may help not only in identifying novel therapeutic strategies for OA management but also relevant targets for early-stage intervention.

## Data Availability

Data and data conceptualization are contained within the article.

## References

[1] Long H., Liu Q., Yin H., Wang K., Diao N., Zhang Y., Lin J., Guo A. (2022). Prevalence Trends of Site-Specific Osteoarthritis From 1990 to 2019: Findings From the Global Burden of Disease Study 2019. Arthritis Rheumatol..

[2] Wong A.Y., Samartzis D., Maher C. (2023). The global burden of osteoarthritis: Past and future perspectives. Lancet Rheumatol..

[3] Nelson A.E. (2024). Multiple joint osteoarthritis (mjoa): What’s in a name?. Osteoarthr. Cartil..

[4] Palazzo C., Nguyen C., Lefevre-Colau M.-M., Rannou F., Poiraudeau S. (2016). Risk factors and burden of osteoarthritis. Ann. Phys. Rehabil. Med..

[5] Dorio M., Deveza L.A. (2022). Phenotypes in osteoarthritis: Why do we need them and where are we at?. Clin. Geriatr. Med..

[6] Deveza L.A., Nelson A.E., Loeser R.F. (2019). Phenotypes of osteoarthritis: Current state and future implications. Clin. Exp. Rheumatol..

[7] Zhuo Q., Yang W., Chen J., Wang Y. (2012). Metabolic syndrome meets osteoarthritis. Nat. Rev. Rheumatol..

[8] Mobasheri A., Rayman M.P., Gualillo O., Sellam J., van der Kraan P., Fearon U. (2017). The role of metabolism in the pathogenesis of osteoarthritis. Nat. Rev. Rheumatol..

[9] Goldring M.B., Goldring S.R. (2007). Osteoarthritis. J. Cell Physiol..

[10] Glyn-Jones S., Palmer A.J., Agricola R., Price A.J., Vincent T.L., Weinans H., Carr A.J. (2015). Osteoarthritis. Lancet.

[11] van Doormaal M.C., Meerhoff G.A., Vlieland T.P.V., Peter W.F. (2020). A clinical practice guideline for physical therapy in patients with hip or knee osteoarthritis. Musculoskelet. Care.

[12] Mao L., Wu W., Wang M., Guo J., Li H., Zhang S., Xu J., Zou J. (2021). Targeted treatment for osteoarthritis: Drugs and delivery system. Drug Deliv..

[13] Cooper C., Chapurlat R., Al-Daghri N., Herrero-Beaumont G., Bruyere O., Rannou F., Roth R., Uebelhart D., Reginster J.Y. (2019). Safety of oral non-selective non-steroidal anti-inflammatory drugs in osteoarthritis: What does the literature say?. Drugs Aging.

[14] da Costa B.R., Pereira T.V., Saadat P., Rudnicki M., Iskander S.M., Bodmer N.S., Bobos P., Gao L., Kiyomoto H.D., Montezuma T. (2021). Effectiveness and safety of non-steroidal anti-inflammatory drugs and opioid treatment for knee and hip osteoarthritis: Network meta-analysis. BMJ.

[15] Liang Y., Xu X., Xu L., Prasadam I., Duan L., Xiao Y., Xia J. (2021). Non-surgical osteoarthritis therapy, intra-articular drug delivery towards clinical applications. J. Drug Target..

[16] Ma L., Zheng X., Lin R., Sun A.R., Song J., Ye Z., Liang D., Zhang M., Tian J., Zhou X. (2022). Knee Osteoarthritis Therapy: Recent Advances in Intra-Articular Drug Delivery Systems. Drug Des. Dev. Ther..

[17] Szwedowski D., Szczepanek J., Paczesny Ł., Zabrzyński J., Gagat M., Mobasheri A., Jeka S. (2021). The Effect of Platelet-Rich Plasma on the Intra-Articular Microenvironment in Knee Osteoarthritis. Int. J. Mol. Sci..

[18] Gan X., Wang X., Huang Y., Li G., Kang H. (2024). Applications of Hydrogels in Osteoarthritis Treatment. Biomedicines.

[19] Farinelli L., Riccio M., Gigante A., De Francesco F. (2024). Pain Management Strategies in Osteoarthritis. Biomedicines.

[20] Roelofs A.J., De Bari C. (2024). Osteoarthritis year in review 2023: Biology. Osteoarthr. Cartil..

[21] Scanzello C.R., Loeser R.F. (2015). Editorial: Inflammatory Activity in Symptomatic Knee Osteoarthritis: Not All Inflammation Is Local. Arthritis Rheumatol..

[22] Berenbaum F. (2013). Osteoarthritis as an inflammatory disease (osteoarthritis is not osteoarthrosis!). Osteoarthr. Cartil..

[23] Loeser R.F., Collins J.A., Diekman B.O. (2016). Ageing and the pathogenesis of osteoarthritis. Nat. Rev. Rheumatol..

[24] Franceschi C., Bonafe M., Valensin S., Olivieri F., De Luca M., Ottaviani E., De Benedictis G. (2000). Inflamm-aging: An evolutionary perspective on immunosenescence. Ann. N. Y. Acad. Sci..

[25] Lopez-Otin C., Blasco M.A., Partridge L., Serrano M., Kroemer G. (2013). The hallmarks of aging. Cell.

[26] López-Otín C., Blasco M.A., Partridge L., Serrano M., Kroemer G. (2023). Hallmarks of aging: An expanding universe. Cell.

[27] Loeser R.F., Goldring S.R., Scanzello C.R., Goldring M.B. (2012). Osteoarthritis: A disease of the joint as an organ. Arthritis Rheum..

[28] Dilley J.E., Bello M.A., Roman N., McKinley T., Sankar U. (2023). Post-traumatic osteoarthritis: A review of pathogenic mechanisms and novel targets for mitigation. Bone Rep..

[29] Martel-Pelletier J., Barr A.J., Cicuttini F.M., Conaghan P.G., Cooper C., Goldring M.B., Goldring S.R., Jones G., Teichtahl A.J., Pelletier J.P. (2016). Osteoarthritis. Nat. Rev. Dis. Primers.

[30] Ilas D.C., Churchman S.M., McGonagle D., Jones E. (2017). Targeting Subchondral Bone Mesenchymal Stem Cell Activities for Intrinsic Joint Repair in Osteoarthritis. Futur. Sci. OA.

[31] Bianco D., Todorov A., Čengić T., Pagenstert G., Schären S., Netzer C., Hügle T., Geurts J. (2018). Alterations of Subchondral Bone Progenitor Cells in Human Knee and Hip Osteoarthritis Lead to a Bone Sclerosis Phenotype. Int. J. Mol. Sci..

[32] Goldring S.R., Goldring M.B. (2016). Changes in the osteochondral unit during osteoarthritis: Structure, function and cartilage–bone crosstalk. Nat. Rev. Rheumatol..

[33] Zeng N., Yan Z.-P., Chen X.-Y., Ni G.-X. (2020). Infrapatellar Fat Pad and Knee Osteoarthritis. Aging Dis..

[34] Paduszynski W., Jeskiewicz M., Uchanski P., Gackowski S., Radkowski M., Demkow U. (2018). Hoffa’s fat pad abnormality in the development of knee osteoarthritis. Adv. Exp. Med. Biol..

[35] Wenham C.Y.J., Conaghan P.G. (2010). The role of synovitis in osteoarthritis. Ther. Adv. Musculoskelet. Dis..

[36] Robinson W.H., Lepus C.M., Wang Q., Raghu H., Mao R., Lindstrom T.M., Sokolove J. (2016). Low-grade inflammation as a key mediator of the pathogenesis of osteoarthritis. Nat. Rev. Rheumatol..

[37] Benito M.J., Veale D.J., FitzGerald O., van den Berg W.B., Bresnihan B. (2005). Synovial tissue inflammation in early and late osteoarthritis. Ann. Rheum. Dis..

[38] Manferdini C., Paolella F., Gabusi E., Silvestri Y., Gambari L., Cattini L., Filardo G., Fleury-Cappellesso S., Lisignoli G. (2016). From osteoarthritic synovium to synovial-derived cells characterization: Synovial macrophages are key effector cells. Arthritis Res. Ther..

[39] Hugle T., Geurts J. (2017). What drives osteoarthritis?-synovial versus subchondral bone pathology. Rheumatology.

[40] Sellam J., Berenbaum F. (2010). The role of synovitis in pathophysiology and clinical symptoms of osteoarthritis. Nat. Rev. Rheumatol..

[41] Carmona-Rivera C., Carlucci P.M., Moore E., Lingampalli N., Uchtenhagen H., James E., Liu Y., Bicker K.L., Wahamaa H., Hoffmann V. (2017). Synovial fibroblast-neutrophil interactions promote pathogenic adaptive immunity in rheumatoid arthritis. Sci. Immunol..

[42] Da R.-R., Qin Y., Baeten D., Zhang Y. (2007). B Cell Clonal Expansion and Somatic Hypermutation of Ig Variable Heavy Chain Genes in the Synovial Membrane of Patients with Osteoarthritis. J. Immunol..

[43] Tu J., Huang W., Zhang W., Mei J., Zhu C. (2021). A Tale of Two Immune Cells in Rheumatoid Arthritis: The Crosstalk Between Macrophages and T Cells in the Synovium. Front. Immunol..

[44] Orlowsky E.W., Kraus V.B. (2015). The Role of Innate Immunity in Osteoarthritis: When Our First Line of Defense Goes On the Offensive. J. Rheumatol..

[45] Kuang G., Tan X., Liu X., Li N., Yi N., Mi Y., Shi Q., Zeng F., Xie X., Lu M. (2024). The Role of Innate Immunity in Osteoarthritis and the Connotation of “Immune-joint” Axis: A Narrative Review. Comb. Chem. High Throughput Screen..

[46] Chen Z., Ma Y., Li X., Deng Z., Zheng M., Zheng Q. (2020). The Immune Cell Landscape in Different Anatomical Structures of Knee in Osteoarthritis: A Gene Expression-Based Study. BioMed Res. Int..

[47] Veglia F., Perego M., Gabrilovich D. (2018). Myeloid-derived suppressor cells coming of age. Nat. Immunol..

[48] Damuzzo V., Pinton L., Desantis G., Solito S., Marigo I., Bronte V., Mandruzzato S. (2015). Complexity and challenges in defining myeloid-derived suppressor cells. Cytom. Part B Clin. Cytom..

[49] Condamine T., Dominguez G.A., Youn J.-I., Kossenkov A.V., Mony S., Alicea-Torres K., Tcyganov E., Hashimoto A., Nefedova Y., Lin C. (2016). Lectin-type oxidized LDL receptor-1 distinguishes population of human polymorphonuclear myeloid-derived suppressor cells in cancer patients. Sci. Immunol..

[50] Ling Z., Yang C., Tan J., Dou C., Chen Y. (2021). Beyond immunosuppressive effects: Dual roles of myeloid-derived suppressor cells in bone-related diseases. Cell. Mol. Life Sci..

[51] Zhang H., Wang S., Huang Y., Wang H., Zhao J., Gaskin F., Yang N., Fu S.M. (2015). Myeloid-derived suppressor cells are proinflammatory and regulate collagen-induced arthritis through manipulating Th17 cell differentiation. Clin. Immunol..

[52] Park M.-J., Lee S.-H., Kim E.-K., Lee E.-J., Baek J.-A., Park S.-H., Kwok S.-K., Cho M.-L. (2018). Interleukin-10 produced by myeloid-derived suppressor cells is critical for the induction of Tregs and attenuation of rheumatoid inflammation in mice. Sci. Rep..

[53] Rajabinejad M., Salari F., Gorgin Karaji A., Rezaiemanesh A. (2019). The role of myeloid-derived suppressor cells in the pathogenesis of rheumatoid arthritis; anti- or pro-inflammatory cells?. Artif. Cells Nanomed. Biotechnol..

[54] Tang C., Liu Q., Zhang Y., Liu G., Shen G. (2021). Identification of CIRBP and TRPV4 as Immune-Related Diagnostic Biomarkers in Osteoarthritis. Int. J. Gen. Med..

[55] Danilin S., Merkel A.R., Johnson J.R., Johnson R.W., Edwards J.R., Sterling J.A. (2012). Myeloid-derived suppressor cells expand during breast cancer progression and promote tumor-induced bone destruction. OncoImmunology.

[56] Edgington-Mitchell L.E., Rautela J., Duivenvoorden H.M., Jayatilleke K.M., van der Linden W.A., Verdoes M., Bogyo M., Parker B.S. (2015). Cysteine cathepsin activity suppresses osteoclastogenesis of myeloid-derived suppressor cells in breast cancer. Oncotarget.

[57] Zhuang J., Zhang J., Lwin S.T., Edwards J.R., Edwards C.M., Mundy G.R., Yang X. (2012). Osteoclasts in Multiple Myeloma Are Derived from Gr-1+CD11b+Myeloid-Derived Suppressor Cells. PLoS ONE.

[58] Sawant A., Ponnazhagan S. (2013). Myeloid-Derived Suppressor Cells as Osteoclast Progenitors: A Novel Target for Controlling Osteolytic Bone Metastasis. Cancer Res..

[59] Kirkwood K.L., Zhang L., Thiyagarajan R., Seldeen K.L., Troen B.R. (2018). Myeloid-Derived Suppressor Cells at the Intersection of Inflammaging and Bone Fragility. Immunol. Investig..

[60] Kwack K.H., Maglaras V., Thiyagarajan R., Zhang L., Kirkwood K.L. (2021). Myeloid-derived suppressor cells in obesity-associated periodontal disease: A conceptual model. Periodontology 2000.

[61] Kwack K.H., Zhang L., Kirkwood K.L. (2024). In vitro osteoclastogenesis assessment using murine myeloid-derived suppressor cells. Methods Cell Biol..

[62] Zhang L., Kirkwood C.L., Sohn J., Lau A., Bayers-Thering M., Bali S.K., Rachala S., Marzo J.M., Anders M.J., Beier F. (2021). Expansion of myeloid-derived suppressor cells contributes to metabolic osteoarthritis through subchondral bone remodeling. Arthritis Res. Ther..

[63] Ziegler-Heitbrock H.L., Passlick B., Flieger D. (1988). The Monoclonal Antimonocyte Antibody My4 Stains B Lymphocytes and Two Distinct Monocyte Subsets in Human Peripheral Blood. Hybridoma.

[64] Passlick B., Flieger D., Ziegler-Heitbrock H.W. (1989). Identification and characterization of a novel monocyte subpopulation in human peripheral blood. Blood.

[65] Ożańska A., Szymczak D., Rybka J. (2020). Pattern of human monocyte subpopulations in health and disease. Scand. J. Immunol..

[66] Guillem-Llobat P., Marín M., Rouleau M., Silvestre A., Blin-Wakkach C., Ferrándiz M.L., Guillén M.I., Ibáñez L. (2024). New Insights into the Pro-Inflammatory and Osteoclastogenic Profile of Circulating Monocytes in Osteoarthritis Patients. Int. J. Mol. Sci..

[67] Lee H.-R., Lee S., Yoo I.S., Yoo S.-J., Kwon M.-H., Joung C.-I., Park J.A., Kang S.W., Kim J. (2022). CD14+ monocytes and soluble CD14 of synovial fluid are associated with osteoarthritis progression. Arch. Rheumatol..

[68] Evers T.M., Sheikhhassani V., Haks M.C., Storm C., Ottenhoff T.H., Mashaghi A. (2021). Single-cell analysis reveals chemokine-mediated differential regulation of monocyte mechanics. iScience.

[69] Goldring M.B., Otero M. (2011). Inflammation in osteoarthritis. Curr. Opin. Rheumatol..

[70] Raghu H., Lepus C.M., Wang Q., Wong H.H., Lingampalli N., Oliviero F., Punzi L., Giori N.J., Goodman S.B., Chu C.R. (2017). Ccl2/ccr2, but not ccl5/ccr5, mediates monocyte recruitment, inflammation and cartilage destruction in osteoarthritis. Ann. Rheum. Dis..

[71] Mondadori C., Palombella S., Salehi S., Talò G., Visone R., Rasponi M., Redaelli A., Sansone V., Moretti M., Lopa S. (2021). Recapitulating monocyte extravasation to the synovium in an organotypic microfluidic model of the articular joint. Biofabrication.

[72] Zhao X., Gu M., Xu X., Wen X., Yang G., Li L., Sheng P., Meng F. (2020). Ccl3/ccr1 mediates cd14(+)cd16(-) circulating monocyte recruitment in knee osteoarthritis progression. Osteoarthr. Cartil..

[73] Wojdasiewicz P., Poniatowski A., Kotela A., Deszczyński J., Kotela I., Szukiewicz D. (2014). The Chemokine CX3CL1 (Fractalkine) and its Receptor CX3CR1: Occurrence and Potential Role in Osteoarthritis. Arch. Immunol. Ther. Exp..

[74] Endres M., Andreas K., Kalwitz G., Freymann U., Neumann K., Ringe J., Sittinger M., Häupl T., Kaps C. (2010). Chemokine profile of synovial fluid from normal, osteoarthritis and rheumatoid arthritis patients: CCL25, CXCL10 and XCL1 recruit human subchondral mesenchymal progenitor cells. Osteoarthr. Cartil..

[75] Fantuzzi L., Tagliamonte M., Gauzzi M.C., Lopalco L. (2019). Dual ccr5/ccr2 targeting: Opportunities for the cure of complex disorders. Cell. Mol. Life Sci..

[76] Ni F., Zhang Y., Peng X., Li J. (2020). Correlation between osteoarthritis and monocyte chemotactic protein-1 expression: A meta-analysis. J. Orthop. Surg. Res..

[77] Loukov D., Karampatos S., Maly M., Bowdish D. (2018). Monocyte activation is elevated in women with knee-osteoarthritis and associated with inflammation, BMI and pain. Osteoarthr. Cartil..

[78] Yunna C., Mengru H., Lei W., Weidong C. (2020). Macrophage M1/M2 polarization. Eur. J. Pharmacol..

[79] Wood M.J., Leckenby A., Reynolds G., Spiering R., Pratt A.G., Rankin K.S., Isaacs J.D., Haniffa M.A., Milling S., Hilkens C.M. (2019). Macrophage proliferation distinguishes 2 subgroups of knee osteoarthritis patients. J. Clin. Investig..

[80] Kurowska-Stolarska M., Alivernini S. (2017). Synovial tissue macrophages: Friend or foe?. RMD Open.

[81] Shi C., Pamer E.G. (2011). Monocyte recruitment during infection and inflammation. Nat. Rev. Immunol..

[82] Luukkonen J., Huhtakangas J., Palosaari S., Tuukkanen J., Vuolteenaho O., Lehenkari P. (2022). Preliminary Report: Osteoarthritis and Rheumatoid Arthritis Synovial Fluid Increased Osteoclastogenesis In Vitro by Monocyte Differentiation Pathway Regulating Cytokines. Mediat. Inflamm..

[83] Hirose S., Lin Q., Ohtsuji M., Nishimura H., Verbeek J.S. (2019). Monocyte subsets involved in the development of systemic lupus erythematosus and rheumatoid arthritis. Int. Immunol..

[84] Monibi F., Roller B.L., Stoker A., Garner B., Bal S., Cook J.L. (2015). Identification of Synovial Fluid Biomarkers for Knee Osteoarthritis and Correlation with Radiographic Assessment. J. Knee Surg..

[85] Gómez-Aristizábal A., Gandhi R., Mahomed N.N., Marshall K.W., Viswanathan S. (2019). Synovial fluid monocyte/macrophage subsets and their correlation to patient-reported outcomes in osteoarthritic patients: A cohort study. Arthritis Res. Ther..

[86] Shi J., Zhao W., Ying H., Du J., Chen J., Chen S., Shen B. (2017). The relationship of platelet to lymphocyte ratio and neutrophil to monocyte ratio to radiographic grades of knee osteoarthritis. Z. Fur Rheumatol..

[87] Gao K., Zhu W., Liu W., Ma D., Li H., Yu W., Wang L., Cao Y., Jiang Y. (2019). Diagnostic value of the blood monocyte–lymphocyte ratio in knee osteoarthritis. J. Int. Med. Res..

[88] Lendeckel U., Venz S., Wolke C. (2022). Macrophages: Shapes and functions. ChemTexts.

[89] Zhao K., Ruan J., Nie L., Ye X., Li J. (2023). Effects of synovial macrophages in osteoarthritis. Front. Immunol..

[90] Murray P.J. (2017). Macrophage polarization. Annu. Rev. Physiol..

[91] Yao Y., Xu X.-H., Jin L. (2019). Macrophage Polarization in Physiological and Pathological Pregnancy. Front. Immunol..

[92] Zhang H., Lin C., Zeng C., Wang Z., Wang H., Lu J., Liu X., Shao Y., Zhao C., Pan J. (2018). Synovial macrophage M1 polarisation exacerbates experimental osteoarthritis partially through R-spondin-2. Ann. Rheum. Dis..

[93] Pessler F., Chen L.X., Dai L., Gomez-Vaquero C., Diaz-Torne C., Paessler M.E., Scanzello C., Çakir N., Einhorn E., Schumacher H.R. (2008). A histomorphometric analysis of synovial biopsies from individuals with Gulf War Veterans’ Illness and joint pain compared to normal and osteoarthritis synovium. Clin. Rheumatol..

[94] Zhang H., Cai D., Bai X. (2020). Macrophages regulate the progression of osteoarthritis. Osteoarthr. Cartil..

[95] van Lent P.L., Blom A.B., van der Kraan P., Holthuysen A.E., Vitters E., van Rooijen N., Smeets R.L., Nabbe K.C., van den Berg W.B. (2004). Crucial role of synovial lining macrophages in the promotion of transforming growth factor beta-mediated osteophyte formation. Arthritis Rheum..

[96] Blom A.B., van Lent P.L., Holthuysen A.E., van der Kraan P.M., Roth J., van Rooijen N., van den Berg W.B. (2004). Synovial lining macrophages mediate osteophyte formation during experimental osteoarthritis. Osteoarthr. Cartil..

[97] Topoluk N., Steckbeck K., Siatkowski S., Burnikel B., Tokish J., Mercuri J. (2017). Amniotic mesenchymal stem cells mitigate osteoarthritis progression in a synovial macrophage-mediated in vitro explant coculture model. J. Tissue Eng. Regen. Med..

[98] Takano S., Uchida K., Inoue G., Miyagi M., Aikawa J., Iwase D., Iwabuchi K., Matsumoto T., Satoh M., Mukai M. (2017). Nerve growth factor regulation and production by macrophages in osteoarthritic synovium. Clin. Exp. Immunol..

[99] Kraus V., McDaniel G., Huebner J., Stabler T., Pieper C., Shipes S., Petry N., Low P., Shen J., McNearney T. (2016). Direct in vivo evidence of activated macrophages in human osteoarthritis. Osteoarthr. Cartil..

[100] Utomo L., Bastiaansen-Jenniskens Y., Verhaar J., van Osch G. (2016). Cartilage inflammation and degeneration is enhanced by pro-inflammatory (M1) macrophages in vitro, but not inhibited directly by anti-inflammatory (M2) macrophages. Osteoarthr. Cartil..

[101] Culemann S., Grüneboom A., Nicolás-Ávila J.Á., Weidner D., Lämmle K.F., Rothe T., Quintana J.A., Kirchner P., Krljanac B., Eberhardt M. (2019). Locally renewing resident synovial macrophages provide a protective barrier for the joint. Nature.

[102] Mocanu V., Timofte D.V., Zară-Dănceanu C.-M., Labusca L. (2024). Obesity, Metabolic Syndrome, and Osteoarthritis Require Integrative Understanding and Management. Biomedicines.

[103] Liu B., Zhang M., Zhao J., Zheng M., Yang H. (2018). Imbalance of m1/m2 macrophages is linked to severity level of knee osteoarthritis. Exp. Ther. Med..

[104] Batoon L., Hawse J.R., McCauley L.K., Weivoda M.M., Roca H. (2024). Efferocytosis and Bone Dynamics. Curr. Osteoporos. Rep..

[105] Luo H., Li L., Han S., Liu T. (2024). The role of monocyte/macrophage chemokines in pathogenesis of osteoarthritis: A review. Int. J. Immunogenetics.

[106] Ziegler-Heitbrock L., Ancuta P., Crowe S., Dalod M., Grau V., Hart D.N., Leenen P.J.M., Liu Y.-J., MacPherson G., Randolph G.J. (2010). Nomenclature of monocytes and dendritic cells in blood. Blood.

[107] Lande R., Giacomini E., Serafini B., Rosicarelli B., Sebastiani G.D., Minisola G., Tarantino U., Riccieri V., Valesini G., Coccia E.M. (2004). Characterization and Recruitment of Plasmacytoid Dendritic Cells in Synovial Fluid and Tissue of Patients with Chronic Inflammatory Arthritis. J. Immunol..

[108] Collin M., Bigley V. (2018). Human dendritic cell subsets: An update. Immunology.

[109] Moret F.M., Hack C.E., van der Wurff-Jacobs K.M., de Jager W., Radstake T.R., Lafeber F.P., van Roon J.A. (2013). Intra-articular cd1c-expressing myeloid dendritic cells from rheumatoid arthritis patients express a unique set of t cell-attracting chemokines and spontaneously induce th1, th17 and th2 cell activity. Arthritis Res. Ther..

[110] Marzaioli V., Canavan M., Floudas A., Flynn K., Mullan R., Veale D.J., Fearon U. (2021). Cd209/cd14(+) dendritic cells characterization in rheumatoid and psoriatic arthritis patients: Activation, synovial infiltration, and therapeutic targeting. Front. Immunol..

[111] E X., Cao Y., Meng H., Qi Y., Du G., Xu J., Bi Z. (2012). Dendritic Cells of Synovium in Experimental Model of Osteoarthritis of Rabbits. Cell. Physiol. Biochem..

[112] Nie F., Ding F., Chen B., Huang S., Liu Q., Xu C. (2019). Dendritic cells aggregate inflammation in experimental osteoarthritis through a toll-like receptor (TLR)-dependent machinery response to challenges. Life Sci..

[113] Segura E., Touzot M., Bohineust A., Cappuccio A., Chiocchia G., Hosmalin A., Dalod M., Soumelis V., Amigorena S. (2013). Human Inflammatory Dendritic Cells Induce Th17 Cell Differentiation. Immunity.

[114] Bertola A., Ciucci T., Rousseau D., Bourlier V., Duffaut C., Bonnafous S., Blin-Wakkach C., Anty R., Iannelli A., Gugenheim J. (2012). Identification of Adipose Tissue Dendritic Cells Correlated With Obesity-Associated Insulin-Resistance and Inducing Th17 Responses in Mice and Patients. Diabetes.

[115] Chou C.-H., Jain V., Gibson J., Attarian D.E., Haraden C.A., Yohn C.B., Laberge R.-M., Gregory S., Kraus V.B. (2020). Synovial cell cross-talk with cartilage plays a major role in the pathogenesis of osteoarthritis. Sci. Rep..

[116] Nefla M., Holzinger D., Berenbaum F., Jacques C. (2016). The danger from within: Alarmins in arthritis. Nat. Rev. Rheumatol..

[117] Corr E.M., Cunningham C.C., Helbert L., McCarthy G.M., Dunne A. (2017). Osteoarthritis-associated basic calcium phosphate crystals activate membrane proximal kinases in human innate immune cells. Arthritis Res. Ther..

[118] Kraus V., Burnett B., Coindreau J., Cottrell S., Eyre D., Gendreau M., Gardiner J., Garnero P., Hardin J., Henrotin Y. (2011). Application of biomarkers in the development of drugs intended for the treatment of osteoarthritis. Osteoarthr. Cartil..

[119] Arden N.B., Cooper C., Guermazi A., Hayashi D., Hunter D., Javaid M.K., Rannou F., Roemer F., Reginsteret J.-Y. (2014). Atlas of Osteoarthritis.

[120] Rollet-Labelle E., Vaillancourt M., Marois L., Newkirk M.M., E Poubelle P., Naccache P.H. (2013). Cross-linking of IgGs bound on circulating neutrophils leads to an activation of endothelial cells: Possible role of rheumatoid factors in rheumatoid arthritis-associated vascular dysfunction. J. Inflamm..

[121] Hsueh M., Zhang X., Wellman S.S., Bolognesi M., Kraus V.B. (2020). Synergistic Roles of Macrophages and Neutrophils in Osteoarthritis Progression. Arthritis Rheumatol..

[122] Haraden C.A., Huebner J.L., Hsueh M.-F., Li Y.-J., Kraus V.B. (2019). Synovial fluid biomarkers associated with osteoarthritis severity reflect macrophage and neutrophil related inflammation. Arthritis Res. Ther..

[123] Wilkinson D.J., Falconer A.M.D., Wright H.L., Lin H., Yamamoto K., Cheung K., Charlton S.H., Arques M.D.C., Janciauskiene S., Refaie R. (2022). Matrix metalloproteinase-13 is fully activated by neutrophil elastase and inactivates its serpin inhibitor, alpha-1 antitrypsin: Implications for osteoarthritis. FEBS J..

[124] Wang G., Jing W., Bi Y., Li Y., Ma L., Yang H., Zhang Y. (2021). Neutrophil Elastase Induces Chondrocyte Apoptosis and Facilitates the Occurrence of Osteoarthritis via Caspase Signaling Pathway. Front. Pharmacol..

[125] Manukyan G., Gallo J., Mikulkova Z., Trajerova M., Savara J., Slobodova Z., Fidler E., Shrestha B., Kriegova E. (2022). Phenotypic and functional characterisation of synovial fluid-derived neutrophils in knee osteoarthritis and knee infection. Osteoarthr. Cartil..

[126] Molnar V., Matišić V., Kodvanj I., Bjelica R., Jeleč Ž., Hudetz D., Rod E., Čukelj F., Vrdoljak T., Vidović D. (2021). Cytokines and Chemokines Involved in Osteoarthritis Pathogenesis. Int. J. Mol. Sci..

[127] Tamassia N., Bianchetto-Aguilera F., Arruda-Silva F., Gardiman E., Gasperini S., Calzetti F., Cassatella M.A. (2018). Cytokine production by human neutrophils: Revisiting the “dark side of the moon”. Eur. J. Clin. Investig..

[128] Benigni G., Dimitrova P., Antonangeli F., Sanseviero E., Milanova V., Blom A., van Lent P., Morrone S., Santoni A., Bernardini G. (2017). Cxcr3/cxcl10 axis regulates neutrophil-nk cell cross-talk determining the severity of experimental osteoarthritis. J. Immunol..

[129] Simon D., Simon H.U. (2007). Eosinophilic disorders. J. Allergy Clin. Immunol..

[130] Anthony R.M., Rutitzky L.I., Urban J.F., Stadecker M.J., Gause W.C. (2007). Protective immune mechanisms in helminth infection. Nat. Rev. Immunol..

[131] Shamri R., Xenakis J.J., Spencer L.A. (2010). Eosinophils in innate immunity: An evolving story. Cell Tissue Res..

[132] Iwaszko M., Biały S., Bogunia-Kubik K. (2021). Significance of Interleukin (IL)-4 and IL-13 in Inflammatory Arthritis. Cells.

[133] Swain S.L., Mckonzie D.T., Dutton R.W., Tonkonogy S.L., English M. (1988). The Role of IL4 and IL5: Characterization of a Distinct Helper T Cell Subset that makes IL4 and IL5 (Th2) and Requires Priming before Induction of Lymphokine Secretion. Immunol. Rev..

[134] Nussbaum J.C., Van Dyken S.J., Von Moltke J., Cheng L.E., Mohapatra A., Molofsky A.B., Thornton E.E., Krummel M.F., Chawla A., Liang H.-E. (2013). Type 2 innate lymphoid cells control eosinophil homeostasis. Nature.

[135] Chen Z., Andreev D., Oeser K., Krljanac B., Hueber A., Kleyer A., Voehringer D., Schett G., Bozec A. (2016). Th2 and eosinophil responses suppress inflammatory arthritis. Nat. Commun..

[136] Finnegan A., Mikecz K., Tao P., Glant T.T. (1999). Proteoglycan (Aggrecan)-Induced Arthritis in BALB/c Mice Is a Th1-Type Disease Regulated by Th2 Cytokines. J. Immunol..

[137] Horsfall A.C., Butler D.M., Marinova L., Warden P.J., O Williams R., Maini R.N., Feldmann M. (1997). Suppression of collagen-induced arthritis by continuous administration of IL-4. J. Immunol..

[138] Cao Y., Brombacher F., Tunyogi-Csapo M., Glant T.T., Finnegan A. (2007). Interleukin-4 regulates proteoglycan-induced arthritis by specifically suppressing the innate immune response. Arthritis Rheum..

[139] Chen Z., Bozec A., Ramming A., Schett G. (2018). Anti-inflammatory and immune-regulatory cytokines in rheumatoid arthritis. Nat. Rev. Rheumatol..

[140] Yamada T., Tani Y., Nakanishi H., Taguchi R., Arita M., Arai H. (2010). Eosinophils promote resolution of acute peritonitis by producing proresolving mediators in mice. FASEB J..

[141] Isobe Y., Kato T., Arita M. (2012). Emerging Roles of Eosinophils and Eosinophil-Derived Lipid Mediators in the Resolution of Inflammation. Front. Immunol..

[142] Evans C.H., Ghivizzani S.C., Robbins P.D. (2022). Osteoarthritis gene therapy in 2022. Curr. Opin. Rheumatol..

[143] Andreev D., Kachler K., Liu M., Chen Z., Krishnacoumar B., Ringer M., Frey S., Krönke G., Voehringer D., Schett G. (2024). Eosinophils preserve bone homeostasis by inhibiting excessive osteoclast formation and activity via eosinophil peroxidase. Nat. Commun..

[144] Meng J., Du H., Lv H., Lu J., Li J., Yao J. (2023). Identification of the osteoarthritis signature gene PDK1 by machine learning and its regulatory mechanisms on chondrocyte autophagy and apoptosis. Front. Immunol..

[145] Moussa K., Gurung P., Adams-Huet B., Devaraj S., Jialal I. (2019). Increased eosinophils in adipose tissue of metabolic syndrome. J. Diabetes Its Complicat..

[146] Liu Y.M., Jiang W.M., Huang J.M., Zhong L.M. (2024). Bioinformatic analysis combined with immune infiltration to explore osteoarthritis biomarkers and drug prediction. Medicine.

[147] Yellin M.J., Winikoff S., Fortune S.M., Baum D., Crow M.K., Lederman S., Chess L. (1995). Ligation of CD40 on fibroblasts induces CD54 (ICAM-1) and CD106 (VCAM-1) up-regulation and IL-6 production and proliferation. J. Leukoc. Biol..

[148] Permin H., Skov P.S., Norn S. (1983). Basophil Histamine Release Induced by Leukocyte Nuclei in Patients with Rheumatoid Arthritis. Allergy.

[149] Urb M., Sheppard D.C. (2012). The Role of Mast Cells in the Defence against Pathogens. PLOS Pathog..

[150] Krystel-Whittemore M., Dileepan K.N., Wood J.G. (2016). Mast Cell: A Multi-Functional Master Cell. Front. Immunol..

[151] Ioan-Facsinay A. (2017). Initiating pain in osteoarthritis (OA): Is it the mast cell?. Osteoarthr. Cartil..

[152] Yu Y., Blokhuis B.R., Garssen J., Redegeld F.A. (2016). Non-IgE mediated mast cell activation. Eur. J. Pharmacol..

[153] Redegeld F.A., Yu Y., Kumari S., Charles N., Blank U. (2018). Non-IgE mediated mast cell activation. Immunol. Rev..

[154] Palmer H.S., Kelso E.B., Lockhart J.C., Sommerhoff C.P., Plevin R., Goh F.G., Ferrell W.R. (2007). Protease-activated receptor 2 mediates the proinflammatory effects of synovial mast cells. Arthritis Rheum..

[155] Klein-Wieringa I.R., de Lange-Brokaar B.J., Yusuf E., Andersen S.N., Kwekkeboom J.C., Kroon H.M., van Osch G.J., Zuurmond A.-M., Stojanovic-Susulic V., Nelissen R.G. (2016). Inflammatory Cells in Patients with Endstage Knee Osteoarthritis: A Comparison between the Synovium and the Infrapatellar Fat Pad. J. Rheumatol..

[156] de Lange-Brokaar B., Ioan-Facsinay A., van Osch G., Zuurmond A.-M., Schoones J., Toes R., Huizinga T., Kloppenburg M. (2012). Synovial inflammation, immune cells and their cytokines in osteoarthritis: A review. Osteoarthr. Cartil..

[157] Nigrovic P.A., Lee D.M. (2007). Synovial mast cells: Role in acute and chronic arthritis. Immunol. Rev..

[158] de Lange-Brokaar B.J., Kloppenburg M., Andersen S.N., Dorjée A.L., Yusuf E., Herb-van Toorn L., Kroon H.M., Zuurmond A.M., Stojanovic-Susulic V., Bloem J.L. (2016). Characterization of synovial mast cells in knee osteoarthritis: Association with clinical parameters. Osteoarthr. Cartil..

[159] Uchida K., Takano S., Inoue G., Iwase D., Aikawa J., Takata K., Tazawa R., Kawakubo A., Sekiguchi H., Takaso M. (2019). Increase in mast cell marker expression in the synovium of obese patients with osteoarthritis of the knee. Diabetes Metab. Syndr. Obes. Targets Ther..

[160] Sousa-Valente J., Calvo L., Vacca V., Simeoli R., Arévalo J., Malcangio M. (2018). Role of TrkA signalling and mast cells in the initiation of osteoarthritis pain in the monoiodoacetate model. Osteoarthr. Cartil..

[161] Pitcher T., Sousa-Valente J., Malcangio M. (2016). The monoiodoacetate model of osteoarthritis pain in the mouse. J. Vis. Exp..

[162] Huss R.S., Huddleston J.I., Goodman S.B., Butcher E.C., Zabel B.A. (2010). Synovial tissue–infiltrating natural killer cells in osteoarthritis and periprosthetic inflammation. Arthritis Rheum..

[163] Wang H., Zeng Y., Zhang M., Ma H., Xu B., Jiang H., Wang J., Li G. (2019). Cd56(bright)cd16(-) natural killer cells are shifted toward an ifn-gamma-promoting phenotype with reduced regulatory capacity in osteoarthritis. Hum. Immunol..

[164] Dogra P., Rancan C., Ma W., Toth M., Senda T., Carpenter D.J., Kubota M., Matsumoto R., Thapa P., Szabo P.A. (2020). Tissue Determinants of Human NK Cell Development, Function, and Residence. Cell.

[165] Vivier E., Tomasello E., Baratin M., Walzer T., Ugolini S. (2008). Functions of natural killer cells. Nat. Immunol..

[166] Mace E.M. (2022). Human natural killer cells: Form, function, and development. J. Allergy Clin. Immunol..

[167] Regis S., Dondero A., Spaggiari G.M., Serra M., Caliendo F., Bottino C., Castriconi R. (2022). miR-24-3p down-regulates the expression of the apoptotic factors FasL and BIM in human natural killer cells. Cell. Signal..

[168] Siew Y.-Y., Neo S.-Y., Yew H.-C., Lim S.-W., Ng Y.-C., Lew S.-M., Seetoh W.-G., Seow S.-V., Koh H.-L. (2015). Oxaliplatin regulates expression of stress ligands in ovarian cancer cells and modulates their susceptibility to natural killer cell-mediated cytotoxicity. Int. Immunol..

[169] Wu J., He B., Miao M., Han X., Dai H., Dou H., Li Y., Zhang X., Wang G. (2022). Enhancing Natural Killer Cell-Mediated Cancer Immunotherapy by the Biological Macromolecule Nocardia rubra Cell-Wall Skeleton. Pathol. Oncol. Res..

[170] Freud A.G., Mundy-Bosse B.L., Yu J., Caligiuri M.A. (2017). The Broad Spectrum of Human Natural Killer Cell Diversity. Immunity.

[171] Björkström N.K., Ljunggren H.-G., Michaëlsson J. (2016). Emerging insights into natural killer cells in human peripheral tissues. Nat. Rev. Immunol..

[172] Cooper M.A., Elliott J.M., Keyel P.A., Yang L., Carrero J.A., Yokoyama W.M. (2009). Cytokine-induced memory-like natural killer cells. Proc. Natl. Acad. Sci. USA.

[173] Kim K.H., Yu H.T., Hwang I., Park S., Park S.H., Kim S., Shin E.C. (2019). Phenotypic and functional analysis of human nk cell subpopulations according to the expression of fcepsilonrigamma and nkg2c. Front. Immunol..

[174] E Belizário J., Neyra J.M., Rodrigues M.F.S.D. (2018). When and how NK cell-induced programmed cell death benefits immunological protection against intracellular pathogen infection. J. Endotoxin Res..

[175] Drvar V.E.D.R.A.N.A., Curko-Cofek B.O.Z.E.N.A., Karleusa L.J.E.R.K.A., Aralica M.E.R.I.C.A., Rogoznica M.A.R.I.J.A., Kehler T.A.T.J.A.N.A., Legovic D.A.L.E.N., Rukavina D.A.N.I.E.L., Laskarin G.O.R.D.A.N.A. (2022). Granulysin expression and granulysin-mediated apoptosis in the peripheral blood of osteoarthritis patients. Biomed. Rep..

[176] Jaime P., Garcia-Guerrero N., Estella R., Pardo J., Garcia-Alvarez F., Martinez-Lostao L. (2017). Cd56(+)/cd16(-) natural killer cells expressing the inflammatory protease granzyme a are enriched in synovial fluid from patients with osteoarthritis. Osteoarthr. Cartil..

[177] Yang J., Fan Y., Liu S. (2022). ATF3 as a potential diagnostic marker of early-stage osteoarthritis and its correlation with immune infiltration through bioinformatics analysis. Bone Jt. Res..

[178] Zhao W., Liu Y., Liu K., Tu F., Zhang C., Wang H. (2021). Synovial fibroblasts regulate the cytotoxicity and osteoclastogenic activity of synovial natural killer cells through the RANKL-RANK axis in osteoarthritis. Scand. J. Immunol..

[179] Mostafa R.E., Salama A.A. (2023). Eplerenone modulates the inflammatory response in monosodium iodoacetate-induced knee osteoarthritis in rats: Involvement of RANKL/OPG axis. Life Sci..

[180] Groh V., Brühl A., El-Gabalawy H., Nelson J.L., Spies T. (2003). Stimulation of T cell autoreactivity by anomalous expression of NKG2D and its MIC ligands in rheumatoid arthritis. Proc. Natl. Acad. Sci. USA.

[181] Malafoglia V., Ilari S., Gioia C., Vitiello L., Tenti M., Iannuccelli C., Cristiani C.M., Garofalo C., Passacatini L.C., Viglietto G. (2023). An observational study on chronic pain biomarkers in fibromyalgia and osteoarthritis patients: Which role for mu opioid receptor’s expression on NK cells?. Biomedicines.

